# Supplementary material for: The causal relationship between the human gut microbiota and pyogenic arthritis: a Mendelian randomization study
Source: Front Cell Infect Microbiol. 2024 Nov 26;14:1452480. doi: 10.3389/fcimb.2024.1452480 (PMC11629706; doi:10.3389/fcimb.2024.1452480)
Supplement: Supplementary file 1 [file DataSheet1.zip › mendelian test/ebi-a-GCST90027730.csv_leave_one_out.pdf]

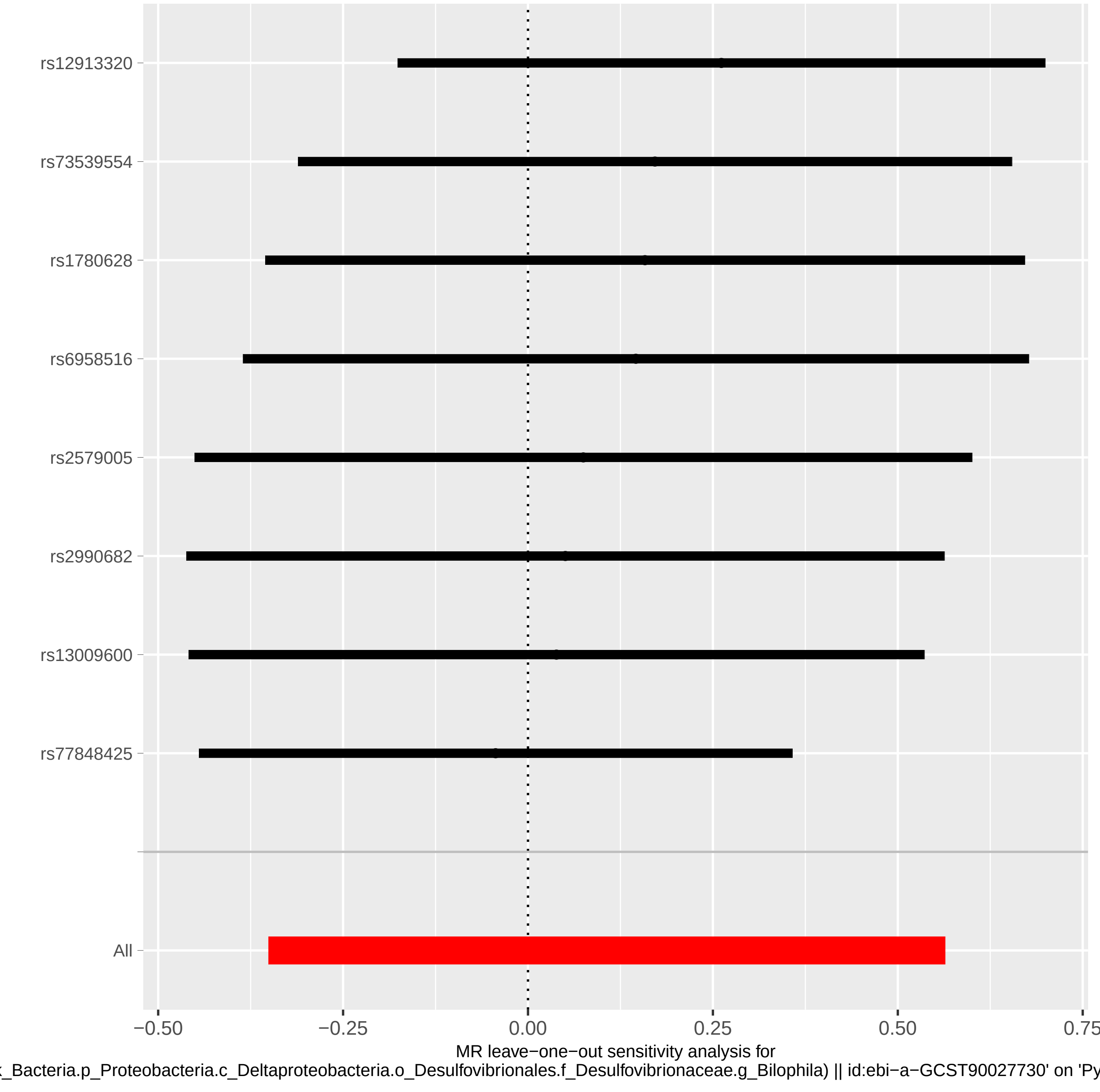

rs12913320
rs73539554
rs1780628
rs6958516
rs2579005
rs2990682
rs13009600
rs77848425
All
−0.50
−0.25
0.00
0.25
0.50
0.75
MR leave−one−out sensitivity analysis for
_Bacteria.p_Proteobacteria.c_Deltaproteobacteria.o_Desulfovibrionales.f_Desulfovibrionaceae.g_Bilophila) || id:ebi−a−GCST90027730' on 'Py
